# Structural, institutional and organizational factors associated with successful pay for performance programmes in improving quality of maternal and child health care in low and middle income countries: a systematic literature review

**DOI:** 10.7189/jogh.08.021001

**Published:** 2018-12

**Authors:** Smruti Patel

**Affiliations:** Independent consultant, Washington, D.C., USA

## Abstract

**Background:**

Pay for Performance (P4P) mechanisms to health facilities and providers have been implemented in several low- and middle-income countries (LMIC) to improve maternal and child health (MCH). These are tied to predetermined quality and quantity indicators. There is limited synthesized information on the structural, institutional and organizational factors that influence the success of P4P programmes with respect to quality of care. This review, which builds on a previously published review sets out to synthesize existing literature on the factors that influence the outcome of P4P programmes and quality of care.

**Methods:**

A literature review was conducted of published studies documenting implementation of P4P interventions and quality of care in low and middle income countries. Records published between June 2014 and September 2017 were selected and combined with articles from January 1990 to June 2014 previously identified by colleagues.

**Results:**

13 studies were included in the final analysis. The majority of studies found a positive impact on quality of care scores and at least one study showed significant reductions in mortality outcomes in newborns. One study from Afghanistan did not show any positive effects. Structural factors associated with likely success of P4P programmes included: explicit acceptance and understanding by health workers; limiting the number of indicators measured with inputs from health workers. Organisational factors included sufficient incentive payments. Notably the main positive outcome identified was facility financial autonomy from additional payments. Verification by external assessors revealed no major manipulation to achieve payment trigger levels. The primary institutional factors identified that P4P programmes fared better when introduced alongside other health reforms and increased funding.

**Conclusions:**

This review has found that P4P is not a uniform intervention, but rather a range of approaches with a substantial variation and complexity in how programmes incorporate quality of care considerations. P4P has shown to have an impact on the quality of a number of limited aspects of maternal and child health care. Further research is needed to understand whether additional aspects of the quality of MCH care could be positively influenced by P4P programmes and how health worker motivation and acceptance are linked to this.

Pay for Performance (P4P) mechanisms to health facilities and providers have been implemented in several low- and middle-income countries (LMIC) to improve maternal and child health (MCH). P4P utilizes financial incentives, and ties payments to health providers or institutions to predetermined quality and quantity indicators. It is critical to understand the key factors that contribute to the successful implementation of P4P programmes. A Cochrane Review conducted in 2012 found the evidence to be too weak to draw conclusions on the effectiveness of P4P to improve the delivery of health interventions in LMIC countries [1]. In addition there is limited synthesized information on the structural, institutional and organizational factors that influence the success of P4P programmes with respect to quality of care. Accordingly, this review, which builds on a previous review conducted by Das and colleagues, sets out to synthesize existing literature on the factors that influence the outcome of P4P programmes and quality of care [2]. First, what are the most frequently cited barriers that could prevent the successful implementation of a P4P programme? Second, are there any key positive factors, cited in the relevant literature that can enable a P4P programme to have a positive effect on quality of care? Even though the specific barriers most relevant for P4P programmes may vary based on context, a comprehensive list of this type will give programme implementers, policymakers, and researchers a synthesized set of factors to consider as they attempt to implement new or improve existing P4P programmes.

## METHODS

### Data sources and searches

A systematic literature review was conducted of published studies documenting implementation of Pay for Performance (alternatively labeled as Performance Based Financing and/or Results Based Financing) interventions and quality of care in low- and middle-income countries. Records were searched in several electronic search engines and databases including MEDLINE, EMBASE, and Web of Science using key words: maternal care, quality of care, antenatal care, emergency obstetric and neonatal care (EmONC) and child care. Additionally, Google Scholar was searched electronically. Websites of key organizations involved in P4P programmes (eg, World Bank, DFID and NORAD) were purposively searched for published articles or working papers. In addition, reference lists from articles and databases were hand searched.

### Study selection

English language studies published between June 2014 and September 2017 from low- and middle-income countries as defined by the World Bank income criteria were included. Study populations comprised of women during pregnancy and post-partum period; children younger than five years; and health workers under assessment for a P4P program. P4P interventions in public or private sector, providing conditional financial incentives to facilities and/or providers to achieve certain performance measures on MCH services including quality were selected. A specific quality score was not calculated. However, studies were assessed for a minimum quality level that was defined as having a control group, randomization and clear description of objectives, interventions, outcomes, power calculations and findings.

### Outcomes of interest

Primary outcome of interest was quality of MCH disaggregated into structural quality, process quality and outcomes. Under structural quality, we considered availability of health facility infrastructure, skilled staff, equipment, commodities, and drugs. For process quality, we included adherence to standard protocols and guidelines for management of health conditions. Morbidity, mortality, out-of-pocket expenses for medical services in the health care facility, and client satisfaction constituted the outcomes.

### Data items and extraction

Country and year of study, study settings and design, sample size, type of incentive (recipient, conditionality and frequency), comparison groups, outcome measures, and quality element of the outcome measures were extracted using a data extraction form.

Identified records published between June 2014 and September 2017 were combined with articles from January 1990 to June 2014 previously identified by Das and colleagues [2].

## RESULTS

**Figure 1:**
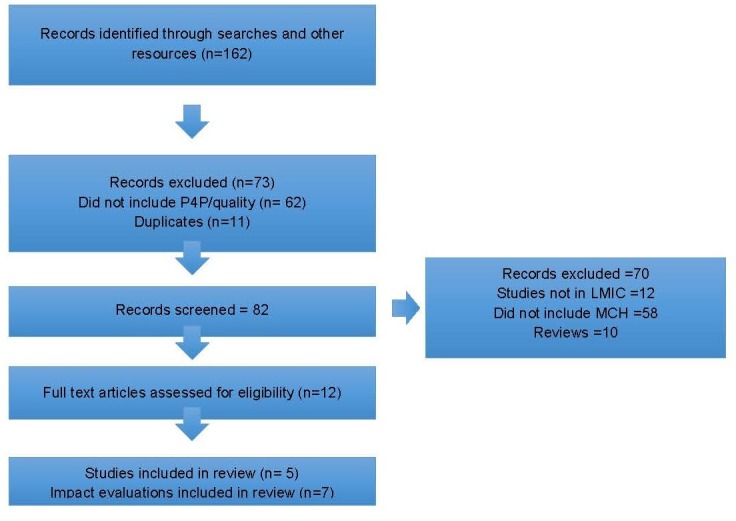
Flow diagram for the selection of articles.

Searches from the databases and others resources identified 155 records. Screened records were 82 after removing duplicates and excluding records that did not mention P4P and quality. From 12 articles eligible for full-text assessment, only 5 were included in the review. Details of the study selection are given in **Figure 1**.

### Study characteristics

**Table 1** outlines the characteristics of the studies included in this review, including those identified by Das [2]. 13 studies (including 8 from Das and colleagues review) and 7 Impact Evaluations of P4P programmes were identified that investigated the effect of P4P on quality of maternal and child care in low- and middle-income countries. These studies indicated that P4P did positively affect the quality of maternal and child care to varying levels.

**Table 1:** Characteristics of the studies included in this review, including those identified by Das [2]

Author, year; Country	Study Design	Program setting	Intervention	Comparison group	Outcome measures	Quality element
Peabody et al, 2011; Philippines [3]	CRT	30 District hospitals (DH)	Bonuses equal to about 5% of a physician’s salary plus system-level incentives that increased compensation to hospitals and across groups of physicians	DHs from matched districts without P4P	Quality of care, utilization of services of children under-five	Process quality
Peabody et al, 2014; Philippines [4]	CRT	30 District hospitals	Bonus payments to physicians if they met qualifying scores on the clinical performance vignette	DHs from matched districts without P4P	Quality of care, utilization of services of children under-five	Clinical outcomes for under-five children
Huillery and Seban 2014; DRC [5]	CRT	152 Facilities (primary and secondary level)	Payments dependent on the verification of declared service volumes at both primary and secondary care levels	Facilities in control districts receiving equivalent fixed payment	User fees, service accessibility, service quality and utilization, population health status, health facility revenue, health workers’ satisfaction, anxiety, motivation	Patient perceived quality and structural quality
Basinga et al, 2011; Rwanda [6]	Controlled before and after	Rural health centers - 80 in intervention and 86 in control	P4P paid directly to facilities and used at their discretion as a supplement to their regular budgets. P4P payments dependent on key MCH outcomes	Facilities under input-based financing received funds equivalent to P4P payments	Prenatal visits, institutional delivery, quality of ANC, child preventive care visits and immunization	Process quality of ANC
Bonfrer et al, 2014; Burundi [7]	Controlled before and after	700 facilities	Based on quantity and quality of services facilities receive performance related funding which on average made up 40% of the facilities budget	Households in the provinces where P4P was not implemented	Utilization and quality of MCH services	Process quality of ANC
Bonfrer et al, 2014; Burundi [8]	Controlled before and after	700 facilities	Based on quantity and quality of services facilities receive performance related funding which on average makes up 40% of the facilities budget	Facilities in control districts receiving normal input financing and salary bonus	Maternal and under-five services	Structural and process quality
Soeters et al, 2011; DRC [9]	Controlled before and after	Two districts	Health facility managers expected to develop business plans, use financial tools to analyze revenues, Facility managers free to negotiate user fees with their communities	Two control districts receiving essential drugs, equipment and fixed staff performance bonuses	Not mentioned	Patient perceived quality, structural and process quality
Huntington et al, 2010; Egypt [10]	Case-control post-test only	Primary health centers	Payments paid according to performance measured against a set of standardized indicators and rating criteria	Primary care providers in control arms got flat rate salary supplements	Quality of ANC, child care services and family planning care	Process quality of ANC, family planning and child care
Gertler. P et al, 2014; Argentina [11]	Controlled before and after	Health facilities	P4P paid based on the provision of quality priority maternal and infant health services to supplement the existing public financing scheme. Health targets are measured using 10 specific indicators derived from best practice clinical protocols	Control clinics were those incorporated later in the same province	Measures of low birthweight, Apgar scores, use of priority services eg, beginning prenatal care in the first 20 weeks of pregnancy, VDRL testing and tetanus vaccines prior to delivery, on-time and complete child immunization, and well-baby visits	Process and clinical outcomes for under-five children
Van de Poel, E et al, 2015; Cambodia [12]	Controlled before and after	Health Facilities	P4P payments for selected services eg, delivery in public facility, vaccinations and antenatal care	Randomly selected districts within same provinces	Measures of child vaccination; antenatal care (at least two visits); delivery in a public facility; and birth-spacing use	Process and clinical outcomes
Engineer CY et al, 2016; Afghanistan [13]	CRT	Primary Care Facilities	P4P bonuses provided to health workers based on volume of 9 health services reported through HMIS plus annual payment based on a balanced scorecard that addresses quality of services and contraceptive prevalence rates	Primary care providers in control arms got flat rate salary	Quality of services including contraception prevalence, skilled deliveries, postnatal visits, vaccinations	Process and clinical outcomes
Talukder N et al, 2015; Bangladesh [14]		Health Facilities	Conditional financial incentives provided to the MNCH team of a health facility for achieving predetermined quantitative and qualitative performance targets	Facilities in same districts as intervention facilities	Quantity and quality of services	Structural and process outcomes
Shen GC et al, 2017; Zambia [15]	Controlled before and after	Health facilities	Bonus payments linked to overall health center performance, and also to individual staff performance. Incentivized payments for nine key health facility indicators found in the HMIS that are deemed as critical to improving maternal and child health services	Districts and facilities in the same province	Job satisfaction, motivation, and attrition	Process
Afghanistan Impact Evaluation Kandpal E; 2016 [16]	Impact Evaluation	Primary care	Facilities were provided a performance bonus of up to ten% of the value of their existing contract with the Government based on a quantity and quality checklist. Additional quality-based payments were made to hospitals but not primary care facilities	Matched facilities in the same province	MCH coverage indicators (modern contraception, antenatal care, skilled birth attendance, postnatal care, and childhood pentavalent vaccination). Quality of patient examinations and counseling, time spent with patients	Process and structural quality, patient perceived quality
Argentina, Impact Evaluation, Kandpal E; 2016 [16]	Impact Evaluation	Facilities	Province-level funding allocated on the basis on beneficiary enrollment as well as providing incentives following a P4P model based on indicators of the use and quality of MCH services and health outcomes	Similar matched districts	Birth outcomes and neonatal mortality	Clinical outcomes
Cameroon, Impact Evaluation, Kandpal E; 2016 [16]	Impact Evaluation	Facilities	The evaluation compared four arms: (1) the standard PBF package, (2) the same level of financing but not linked to performance, and with the same levels of supervision, monitoring, and autonomy as PBF, (3) no additional resources or autonomy, but the same levels of supervision and monitoring as PBF, and (4) pure comparison	Similar matched districts	Vaccinations, family planning, ANC, # of qualified health workers, client satisfaction	Structural and process quality
Democratic Republic of Congo, Impact Evaluation, Kandpal E; 2016 [16]	Impact Evaluation	Facilities	Facility payment determined by the quantity of services provided relative to the other health facilities rather than to the quality of care provided. In contrast, the amount allocated to each facility in the comparison group was calculated based on the staff in the facility.	Similar matched facilities		Process and structural quality, patient perceived quality
Rwanda, Impact Evaluation, Kandpal E; 2016 [16]	Impact Evaluation	Community	(i) demand-side in-kind incentives for women, (ii) performance-based payment for community health worker (CHW) cooperatives, and (iii) combined demand-side and CHW cooperative performance payments	Similar sub districts	Skilled facility births, ANC, PNC, self reported behaviours of CHW (number of hours spent on health work, number of households visited etc.)	Process and clinical outcomes
Zambia, Impact Evaluation, Kandpal E; 2016 [16]	Impact Evaluation	Facilities	three-arm evaluation that tested RBF against an enhanced financing-only arm and a pure comparison arm.	Similar districts	Institutional deliveries, vaccinations, ANC, PNC, health worker satisfaction and motivation	Structural and process quality
Zimbabwe, Impact Evaluation, Kandpal E; 2016 [16]	Impact Evaluation	Facilities	portion of financing received by health facilities depends on the quantity and quality of services, with a focus on maternal and child health.			Structural quality and clinical outcomes

P4P – pay for performance, DH – district hospital, MCH – maternal and child health, HMIS – health management information system, CHW – community health worker, ANC – antenatal care, PNC – postnatal care, RBF – results-based financing

**Table 2** outlines the key findings from studies included in this review.

**Table 2:** Key findings from studies included in the review

Author, year; country	Quality element	Quality outcome measure	Effect size
Peabody et al, 2011; Philippines [3]	Process quality	Provider clinical Mean Vignette score for child health	9.7 percentage points increase
Peabody et al, 2014; Philippines [4]	Clinical outcomes for under-five children	Children underweight for height following discharge from hospital for diarrhea and pneumonia	9 percentage point improvement
Huillery and Seban 2014; DRC [5]	Structural and process quality	Health worker completes consultation report	16 percentage point increase
		Staff attendance	7 percentage point increase
		Perceived health worker workload	16 percentage point decrease
Basinga et al, 2011; Rwanda [6]	Process quality of ANC	Any prenatal care	0.2 percentage point increase
		>4 prenatal care visits	4.4 percentage point increase
		Institutional delivery	23.2 percentage point increase
		Tetanus vaccine during prenatal visit	7.2 percentage point increase
Bonfrer et al, 2014; Burundi [7]	Process quality of ANC	BP measured at least once in pregnancy	6 percentage point increase
		Likelihood of receiving 1 or more anti-tetanus vaccine	10 percentage point increase
		Child being fully vaccinated	4 percentage point increase
Bonfrer et al, 2014; Burundi [8]	Structural and process quality	Women delivering in an institution	22 percentage point increase
		Women using modern family planning services	5 percentage point increase
		Total quality score in clinics	17 percentage point increase
		Felt cured	9 percentage point increase
Soeters et al 2011; DRC [9]	Patient perceived quality, structural and process quality	Patient-perceived availability of drugs	37 percentage point increase
		Patient-perceived quality	15 percentage point increase
		Respect for patients by health facility staff	12 percentage point increase
		Patient perception of being cured	11 percentage point increase
Huntington et al, 2010; Egypt [10]	Process quality of ANC, family planning and child care	Asked parity during ANC visit	12 percentage point increase, *P* < 0.01
		Asked about past illness during ANC visit	32 percentage point increase, *P* < 0.01
		Examined blood pressure during ANC visit	10.2 percentage point increase *P* < 0.05
		Children received follow-up	6.6 percentage point increase *P* < 0.05
		Children explained medication	7.8 percentage point increase *P* < 0.05
		Women knew medicine use in prenatal period	<0.05
Gertler. P et al, 2014; Argentina [11]	Process and clinical outcomes for under-five children	Number of prenatal care visits	6.8 percentage point increase
		Tetanus toxoid	5.6 percentage point increase
		C Section	-5.2 percentage point reduction
		Probability of low birthweight	1.4 percentage point increase
		Neonatal mortality	74% reduction
Van de Poel, E et al, 2015; Cambodia [12]	Process outcomes	Delivery in public facility	6.8 percentage point increase
		Antenatal care	3 percentage point increase
		Vaccination	2.3 percentage point increase
Engineer CY et al, 2016; Afghanistan [13]	Structural and process outcomes	Current use of modern family planning method	-0.5 percentage point reduction
		At least one antenatal checkup by a skilled provider	-0.4 percentage point reduction
		Skilled birth attendant present at latest delivery	5.4 percentage point increase
		Postnatal check up within 42 d of delivery by a skilled provider	0.9 percentage point increase
		Children received pentavalent 3 vaccination	-2.7 percentage point reduction
Talukder N et al, 2015; Bangladesh [14]	Structural and process outcomes	Volume of MCH services	14 percentage point increase
		Changes in quality of MNCH services	26 percentage point increase
Shen GC et al, 2017; Zambia [15]	Health worker Outcomes	Personal well-being	2.42 percentage point increase
		Job satisfaction	4.75 percentage point increase
Kandpal E. Afghanistan, Impact Evaluation; 2016 [16]	Structural and process outcomes		This evaluation was based on the same programme in Afghanistan as that in the paper by Engineer and findings were consistent
Kandpal E. Argentina, Impact Evaluation; 2016. [16]	Clinical outcomes		This evaluation was based on the same programme in Afghanistan as that in the paper by Engineer and findings were consistent
Kandpal E. Cameroon, Impact Evaluation; 2016. [16]	Structural and process quality	Patient satisfaction	8.6 percentage point increase, *P* = 0.077
		Availability of equipment	10.0 percentage point increase, *P* < 0.05
Kandpal E. Democratic Republic of Congo Impact Evaluation; 2016. [16]	Process and structural quality, patient perceived quality	Provision of preventive sessions	43 percentage point increase
		Technical quality of health services	No difference found
		Patient satisfaction	No difference found
		Job satisfaction	14 percentage points lower
		Health workers feeling they have too much work	28% percentage points lower
Kandpal E. Rwanda Impact Evaluation; 2016. [16]	Process and clinical outcomes	Institutional deliveries	Large and significant positive impact
		Quality of prenatal care	Large and significant positive impact
		Utilization of preventative care for young children	Large and significant positive impact
Kandpal E. Zambia Impact Evaluation; 2016. [16]	Structural and process quality	Infrastructure index	Impact estimate 0.483, *P* = 0.099
		Drug availability index	Impact estimate 0.06, *P* = 0.893
		Institutional delivery	12.2% percentage point increase
		Postnatal care	7.8 percentage point increase
		Sufficient time spent with patients	Impact estimate 0.08, *P* = 0.081
Kandpal E. Impact Evaluation; 2016. [16]	Structural quality and clinical outcomes	Delivery by skilled provider	15 percentage point increase, *P* = 0.002
		Delivery in a facility	13 percentage point increase, *P* = 0.003
		Any PNC	11.6 percentage point increase *P* = 0.059
		Use of any contraception	Impact estimate 0.035, *P* = 0.379
		Immunisation all vaccines aged 12-23 mo	Impact estimate 0.003, *P* = 0.978

BP – blood pressure, MCH – maternal and child health, MNCH – maternal, neonatal and child health, ANC – antenatal care, PNC – postnatal care

Many of the studies found positive effects. For example, in P4P districts in Afghanistan providers spent more time with patients; conducted a more complete history and examination and provided more counseling [13]. The Philippines demonstrated a 7%-9% improvement in General Self Reported Health and age adjusted wasting over time in the P4P group. Authors estimated the large impact of higher quality care with 294 cases of wasting averted and 229 more children reporting at least good health [1]. Talukder et al. found average quality of care scores to be higher in the intervention sites, and that the visits conducted by the quality assurance groups acted as refresher trainings for the providers [14]. In Burundi, both the average quality score and the number of women having institutional deliveries increased significantly in the P4P group [7]. In addition patients’ chance of feeling cured was higher under P4P programme in Burundi [8]. Van de Poel also estimated that deliveries increased in a public facility by 7.5% [12]. Significant improvements in the P4P group were also seen for institutional deliveries and preventative care visits for child in Rwanda [6].

The Plan Nacer programme in Argentina demonstrated a significant positive effect on increasing prenatal visits and provision of tetanus toxoid as well as a very significant reduction in neonatal mortality (74%) in the beneficiary group. Interesting, there was also a positive spillover effect with an overall 22% reduction in neonatal mortality (beneficiaries and non-beneficiaries) using the same clinics [11]. In the DRC, 5 out of 6 indicators related to patient perception of quality improved in the P4P sites, some significantly [9].

On the other hand, some studies found that P4P did not show any demonstrable effect on certain indicators. For example, in Afghanistan there was found to be no difference in improving skilled birth attendance or postnatal coverage between intervention and control districts [13]. In addition, the study in Burundi did not find any effect on the use of vaccinations or modern family planning. In Cambodia, P4P did not have a significant effect on antenatal care or vaccination [12].

Overall, the studies revealed the following key elements that contribute to the successful impact of P4P on quality of maternal and child-care.

### Structural factors

#### Perception and acceptance of P4P by health workers

3 studies included in this review discussed the importance of health worker attitudes towards P4P with the literature indicating the need for consultation with, and buy in from the health workers in order for the programme to have an impact. In Egypt, Huntingdon et al. conducted interviews with physicians in the Primary Health Care Units and the district health care officers where the P4P scheme was implemented. The results revealed mixed feedback on the design and functioning of the incentive payment scheme. Healthcare providers voiced concerns that national level decision makers without consulting local administration selected indicators and that too many indicators were used to calculate incentives. There were also problems with delays in receiving incentives that created an atmosphere of distrust and uncertainty [10]. In another study conducted in the DRC, health workers from the P4P group complained about the P4P system and the frustration they had from the inefficiency of their strong efforts to increase the demand - “If there is no patient, we can't do more than working 26 days” [5].

In Afghanistan, when health workers were surveyed only 37.9% in the P4P sites recognized that they had received any payment for P4P intervention even though 86.7% of the P4P health facilities reported that they had received performance payments [13].

Consultation with the health providers on the identification of suitable indicators, transparency on how incentives will be calculated and timely disbursement of payments would result in clearer understanding and ownership with the potential of improved quality of care outcomes.

#### Health worker motivation

It has been suggested that P4P would lead to improved quality of care by motivating health care providers. Of the 13 studies included in this review half considered aspects of health worker motivation and its impact on quality of care within a P4P programme. The evidence from these studies does not necessarily support the view that motivated health care workers will deliver better quality of care. Indeed the literature indicates there is a more complex relationship between incentives and motivation. Engineer and colleagues in Afghanistan suggest that the linkages between payment and motivation of workers to improve targeted services require more finely-tuned understanding of human motivation, as well as more sophisticated approaches to managing organizations and individuals beyond performance payments (eg, taking into account organizational culture, leadership, management and psychology, among other things) [13]. In the DRC, it was found that the introduction of financial incentives led to concrete changes in health workers behaviors. For example, health workers were found to be present at the health facility more often, they organised more preventive health sessions at the facility and conducted more community outreach to sensitize the population on the services offered [5]. The study in Rwanda found similar results, the incentive payment gave providers the motivation to translate their prenatal care knowledge into better practice [6]. Another study has also demonstrated the positive effect of measuring quality without incentives, whereby the act of measurement and feedback in itself led to improvement from awareness and consequent motivation to perform better [3,4].

Examples of motivational outcomes from 3 studies are summarized in **Table 3**.

**Table 3:** Examples of motivational outcomes from 3 studies

Supervision of, feedback to and motivation of health workers	Study
Approximately 50% of providers in the intervention districts reported the benefits of teamwork to ensure appropriate distribution of responsibilities as well as to improve quality of care compared to only 6% in the control districts. Health providers in the intervention districts were twice as likely to receive periodic supervisory visits.	Talukder et al, 2015 [14]
No difference found in indices for motivation and job satisfaction in either the intervention or the control group. The level of performance of health workers was not communicated back to them in either group	Engineer et al, 2016 [13]
PBF schemes brought about a significant increase in job satisfaction and a decrease in attrition, but had no significant effect on motivation.	Shen et al, 2017 [15]

PBF – performance-based financing

#### Indicators and quality measures

It is vitally important to identify indicators and quality measures that are meaningful, measurable and based on best practice clinical protocols. The types and numbers of quality indicators measured varied in the studies identified and included quantitative and qualitative outcomes.

Some examples of quantitative measures include: volume of services, child vaccination rates, contraceptive prevalence rates, institutional delivery rates, prevalence of low birthweight, neonatal mortality, wasting, use of priority services such as beginning antenatal care within first 20 weeks of pregnancy. Qualitative measures included patient satisfaction and health worker satisfaction and motivation.

**Table 4** represents examples of some of the quality indicators measured in the various studies.

**Table 4:** Examples of quality indicators used in the various studies

Quality indicator	Study
Used balanced scorecard with 20 indicators at the health facility level.	Engineer et al, 2016 [13]
Measured age adjusted wasting and general self-reported health measure (GHRH).	Peabody et al, 2014 [4]
Quality Assessment Groups (QAG) comprising of obstetrician, pediatrician and anesthesiologist used web based automated checklists.	Talukder et al, 2015 [14]
Used 10 specific indicators derived from best practice clinical protocols	Gertler et al, 2014 [11]
Quality score comprised of 57 items	Bonfrer et al, 2014 [7,8]
4 specified performance targets: child vaccination; antenatal care (at least two visits); delivery in a public facility; birth-spacing use.	Van de Poel et al, 2015[12]
53 qualitative indicators plus indicators related to patients perception of quality	Soeters et al, 2011 [11]
14 key maternal and child health care output indicators	Basinga et al, 2011 [9]
Curative, preventative and quality of care indicators	Huntington et al, 2010 [12]

### Organisational factors

#### Monitoring and verification

The majority of the studies in this review (9 out of 12) examined the need for monitoring and verification within P4P programmes. Measuring change in quality can be difficult, time consuming, costly and subjective. To overcome these challenges, Peabody and colleagues in the Philippines found Clinical Performance Vignettes to be valuable tools as they provide a detailed measure of the clinical encounters they capture [3,4].

Independent assessors are vital to ensuring verification of data. In Afghanistan the monitoring and verification systems used were quite comprehensive. The total amount of financial incentive paid was adjusted by a quality score based on a National Monitoring Checklist (NMC), which was assessed quarterly by an independent team of provincial officers and consisted of items related to equipment functionality, drug availability, quality of medical charts and number of households visited by Community Health Workers. Health facilities submitted monthly reports on the volume of services provided, which were verified quarterly by independent monitors, record-matching and random home visits of patients reported as service users. Systematic audits of 1100 Health Facility visits verified over 95% of the medical records used for payments, and random sampling of over 29 000 household visits based on medical records verified 89% of the reported services. The community and Health Management Information System verification analysis suggested that there was no major manipulation of the payment triggers by the health facilities, suggesting that the reporting of results for payments was likely to be largely genuine [13]. In Van de Poel’s study there also was no evidence of over reporting in response to financial incentives [12].

Other researchers also utilised independent and blinded assessors in their studies [4,9]. These assessors and interviewers as well as being independent required specific training to ensure adequate capacity to assess quality [4,7].

In Argentina’s Plan Nacer payment of financial incentives were divided with 60% of the maximum payment disbursed monthly based on the number of verified registered beneficiaries; and up to 40% of the maximum transferred every 4 months after verification and certification that the province actually met the quality targets [11].

Results from Egypt are suggestive that care providers do respond to incentives but they must be carefully integrated into a well-known and established quality of the care monitoring system [10].

#### Financial incentive arrangements

The studies in the review revealed a number of financial incentive arrangements from bonuses directed towards individual doctors [3,4] to incentives paid directly to facilities [5] or provinces [11].

In Rwanda, Basinga [6] and colleagues demonstrated larger effects on services for which facilities receive larger financial incentives and those over which the provider has greater control (eg, prenatal care quality and tetanus vaccination during a prenatal care visit) and are less dependent on patients’ health-seeking behaviour (eg, timely prenatal care visits). This finding was supported by Van de Poel [12] who hypothesied that this was likely to be due to the marginal cost of finding and convincing pregnant women to come for regular check-ups that is high compared with the small monetary incentive. On the other hand where the financial incentives was higher, for example for institutional delivery (particularly when implemented as a per case payment) and the health worker had to exert less effort (it is easier to encourage women who have already come into contact with the facility to give birth in it) the impact of the incentive was greater [7].

Huillery and Seban in the DRC also noted that the autonomy of payment allocation among facility staff in the P4P group led to a more egalitarian distribution of payments among workers [5]. P4P benefitted non-technical workers (pharmacists, managers, secretaries, receptionists and maintenance workers) who are not in the governmental payroll and therefore do not receive a share of the fixed payment but who can all contribute to the quality of child and maternal care. Health Facility managers in Afghanistan distributed the performance incentives in a range of ways, which included giving individual bonuses proportional to the health worker’s salary, giving them in equal amounts to all staff, or giving them based on their determination of an individual’s contribution [13].

As seen in the study from Afghanistan, other aspects that were considered in the provision of incentives were baseline conditions and expected improvements. The NGOs delivering the services negotiated with the MOPH to adjust their payment to account for the differences in insecurity and geographical inaccessibility that varied by facility [12].

The size of the incentives paid for services varied between studies, for example in Afghanistan the bonus amounts paid were initially about 6%-11% above the base salary, and increased to about 14%-28%, depending on the health worker’s cadre [13]. In the Philippines the bonuses were equal to about 5 percent of a physician’s salary [3,4].

Countries allocated different incentive amounts to various services. In Rwanda, the highest payment was for institutional deliveries (US$ 4.59), whereas the payment rate for an initial prenatal visit was only US$ 0 · 09 [8]. Argentina utilised a different approach by equally dividing the performance payment among ten indicators, with 4% assigned to each, totaling up to 40%. If the target is met, the province receives the full 40% percent of the capita for that indicator. If it does not meet the target, it receives nothing for that indicator [11].

Performance related payments were generally made every four months [11,13].

The methods by which the total incentivised payment amount was calculated varied in study sites. For example, in Burundi the total payment to a facility was calculated as a weighted sum of the number of provided services in the previous 3 months times their unit payment multiplied by the quantity bonus, which ranged between 1 and 1.25 depending on the score obtained from evaluation of facilities [7,8].

Peabody and colleagues perceived that quality effects seen with incentives provided to individuals may also be possible through indirect financial incentives that operate at the system level. These effects on quality affected performance earlier and to a greater degree than measurement and feedback of performance alone [3].

### Institutional factors

#### Country context

The extent to which the P4P scheme actually had on the improved quality of care has to be viewed within the economic, policy and overall context of the country. 4 studies in this review cited specific contextual issues. Basinga and colleagues in Rwanda note that the P4P scheme was implemented in the context of a larger health sector reform [6]. In the Philippines it is important to note at the time of the study, the increase in the prevalence of wasting was due to severe weather disturbances (hurricanes) in 2006 that affected food supply, shelter, and infrastructure and led to outbreaks of waterborne diseases [4]. In Cambodia and Burundi, the introduction of P4P schemes, as in most other contexts, was accompanied by an increase in budgets [7,12].

## DISCUSSION

This review reports the synthesized findings from 13 studies and 7 Impact Evaluations on the structural, institutional and organizational factors associated with successful P4P programmes in improving quality of maternal and child health care in low and middle income countries.

In general, the review suggests that P4P approaches to health delivery can be effective at improving both coverage and quality of targeted maternal and child health services. However, the improvements achieved are not uniform and can be seen in coverage of preventive services in some programmes and for some conditions but not others.

There has been concern that P4P programmes may negatively affect outcomes that are not incentivized. Most of the studies in this review did not address this issue. However, a recent Impact Evaluation of P4P programme in Zimbabwe [16] found that none of the non-incentivised services investigated showed a decline in the number of cases treated, which would be the case if task shifting was occurring and affecting these services.

The perception and acceptance of P4P programmes by health workers needs careful consideration during planning and implementation. Early consultation with health workers regarding which indicators are to be measured and how the incentive will be calculated could prevent issues seen in Egypt where health workers expressed frustration at having these decisions made at the National level [10]. In addition, the overall number of indicators measured needs to be carefully considered and should cover all aspects of quality and not focus on structural quality as was found in the review conducted by Gergen [17]. Checklists seem to increase in length with time [18]; deliberate review of checklists is required to prevent them becoming too long and cumbersome. Lack of understanding can undermine the potential impact of P4P programme by limiting the behavioural response of health workers. In addition, clear communication about the structure of P4P programmes to health workers will likely improve the acceptance of them. In this regard, careful thought should be given to select indicators that will be acceptable to providers but can also maximize the efficiency of spending.

Many researchers have investigated health worker motivation and there is evidence showing that direct incentives such as P4P as well as organisational incentives such as supervision combined with institutional rewards or punishments do lead to improved quality of care [19]. Qualitative work conducted as a part of the Impact Evaluation of Afghanistan showed that the P4P programme was a good motivator even though salaries and incentives were not always received on time [16]. Studies in this review in DCR and Rwanda reported that incentives improved motivation resulting in higher health worker presence at facilities; more facility based preventative sessions and more community outreach [5,6]. However, of concern are findings from Zimbabwe’s Impact Evaluation where health workers despite being motivated by incentives expressed their dissatisfaction with the size of incentive relative to their tasks and overall higher workload. This may result in a decline in effect of the incentive as time progresses [16].

Financial arrangements for incentive payments were varied, both in size and recipient. One of the main positive outcomes identified was the autonomy provided to facilities by some programmes (Argentina, DRC, and Zimbabwe). These countries welcomed the ability to distribute the incentive payments in an egalitarian manner among facility staff as well as being able to better allocate scarce resources to best suit their needs [11,16]. The health facility is then able to utilise the incentives to address broader health systems challenges such as drug availability.

Another obvious but important aspect is the need to have adequate levels of incentives or else there may be a limit to the possible gains that can be achieved through P4P programmes as health workers may not feel the added effort is worth the reward. In Misiones province of Argentina the strongest evidence for sustained impact from P4P was seen with a substantial 3-fold increase in incentives [16].

Demand-side incentives need to also be considered in a P4P programme as they can work alongside supply-side incentives. The increase in health seeking behaviour, allows more opportunity for health workers to provide quality care and ultimately impact maternal and child health outcomes. The Impact Evaluation in Afghanistan identified the lack of attention to demand-side considerations as one of the flaws of the RBF pilot implemented there [16].

As seen in the studies from Rwanda, Cambodia and Burundi, P4P programmes have often been introduced alongside other health reforms and increased funding. The results from the HRITF Impact Evaluations suggest that P4P programmes should indeed be part of broader health system reforms and complementary intervention. The programmes can be seen as entry points in tackling wider systems issues.

Monitoring and verification is essential to ensure quantity and quality objectives are being met. Feeding performance data back to providers facilitates performance improvement. The Impact Evaluations reviewed establishes the importance of continued innovations on ways to intelligently measure and incentivize quality measures of care in maternal and child health, which are more complex than coverage indicators. It is suggested that the ‘easier’ structural quality indicators are addressed first and then programmes can move onto introducing process measures of clinical care. This will allow health providers to address less complex quality of care issues first, develop better understanding of RBF and quality of care, and then shift gradually toward more demanding measures of care under the RBF programmes [16].

### Limitations

The focus on examining the quality of maternal and child health care is relatively recent and hence there are only a limited number of published articles. This review examined primarily peer reviewed articles. A limitation of this review is not having access to unpublished findings.

## CONCLUSIONS

This review has found that P4P is not a uniform intervention, but rather a range of approaches.

There is substantial variation and complexity in how programmes incorporate quality of care considerations. There are differences in how quality is included in the payment formula, how many and what indicators are utilised in checklists, and how they are measured.

P4P has shown to have an impact on the quality of a number of limited aspects of maternal and child health care and supports the findings of Das and colleagues [2]. In addition to previous findings of an increase in prenatal visits, provision of antenatal tetanus toxoid, institutional deliveries and preventative visits for children aged under 5, a significant reduction in neonatal mortality was found. Patient experience is not a common performance criteria measured though where is has been studied it has been reported to be positively impacted by P4P programmes.

Many of the P4P programmes have some documented or perceived positive spillover effects on individual provider activity and the health system as a whole. From the literature examined, improved generation and use of data are possibly the most important positive spillover effect of the P4P programmes.

Further research is needed to understand whether additional aspects of the quality of maternal and child health care could be positively influenced by P4P programmes and how health worker motivation and health worker acceptance are linked to this.
